# Surveillance Web System and Mouthwash-Saliva qPCR for Labor Ambulatory SARS-CoV-2 Detection and Prevention

**DOI:** 10.3390/ijerph19031271

**Published:** 2022-01-24

**Authors:** Gustavo Mora-Aguilera, Verónica Martínez-Bustamante, Gerardo Acevedo-Sánchez, Juan J. Coria-Contreras, Eduardo Guzmán-Hernández, Oscar E. Flores-Colorado, Coral Mendoza-Ramos, Gabriel Hernández-Nava, Ikuri Álvarez-Maya, M. Alejandra Gutiérrez-Espinosa, Raael Gómez-Linton, Ana Carolina Robles-Bustamante, Alberto Gallardo-Hernández

**Affiliations:** 1 Laboratory of Epidemiological Risk Analysis (LANREF), Montecillo Campus, Postgraduate College, Texcoco 56230, CP, Mexico; bustamanteveronica18@gmail.com (V.M.-B.); geraracevedo@gmail.com (G.A.-S.); juanjosecoria8@gmail.com (J.J.C.-C.); guzman.h.eduardo@gmail.com (E.G.-H.); flores.eder.93@gmail.com (O.E.F.-C.); coralmendozaramos@gmail.com (C.M.-R.); gahna08@gmail.com (G.H.-N.); alexge@colpos.mx (M.A.G.-E.); raaelg@yahoo.com (R.G.-L.); 2 Center for Research and Applied Technology in Jalisco (CIATEJ), Jalisco 44270, CP, Mexico; ikuri.alvarez@gmail.com; 3 Secretary of Health-CDMX, Mexico City 06900, CP, Mexico; dracarolinaroblesbte@gmail.com (A.C.R.-B.); albgallardo@yahoo.com.mx (A.G.-H.)

**Keywords:** coronavirus, monitoring, prevention, surveillance, COVID-19

## Abstract

This study provides a safe and low-cost in-house protocol for RT-qPCR-based detection of SARS-CoV-2 using mouthwash–saliva self-collected specimens to achieve clinical and epidemiological surveillance in a real-time web environment applied to ambulatory populations. The in-house protocol comprises a mouthwash–saliva self-collected specimen, heat virus inactivation, and primers to target virus N-gene region and the human RPP30-gene. Aligning with 209 SARS-CoV-2 sequences confirmed specificity including the Alpha variant from the UK. Development, validation, and statistical comparison with official nasopharyngeal swabbing RT-qPCR test were conducted with 115 specimens of ambulatory volunteers. A web–mobile application platform was developed to integrate a real-time epidemiological and clinical core baseline database with mouthwash–saliva RT-qPCR testing. Nine built-in algorithms were generated for decision-making on testing, confining, monitoring, and self-reports to family, social, and work environments. Epidemiological and clinical follow-up and SARS-CoV-2 testing generated a database of 37,351 entries allowing individual decision-making for prevention. Mouthwash–saliva had higher sensitivity than nasopharyngeal swabbing in detecting asymptomatic and mild symptomatic cases with 720 viral copy number (VCN)/mL as the detection limit (Ct = 37.6). Cycling threshold and viral loading were marginally different (*p* = 0.057) between asymptomatic (35 Ct ± 2.8; 21,767.7 VCN/mL, range 720–77,278) and symptomatic (31.3 Ct ± 4.5; 747,294.3 VCN/mL, range 1433.6–3.08 × 10^6^). We provided proof-of-concept evidence of effective surveillance to target asymptomatic and moderate symptomatic ambulatory individuals based on integrating a bio-safety level II laboratory, self-collected, low-risk, low-cost detection protocol, and a real-time digital monitoring system. Mouthwash–saliva was effective for SARS-CoV-2 sampling for the first time at the community level.

## 1. Introduction

SARS-CoV-2 detection protocols were developed soon after the COVID-19 outbreak on 31 December 2019 [1,2,3]. The main focus was to achieve sensitivity and specificity to discriminate SARS-CoV-2 from other respiratory viruses using RT-qPCR assays [1,3]. Further protocols, based on nasopharyngeal and/or oropharyngeal (NPS/OPS) swabs and sputum specimens, adhere to the same principles using up to four gene targets and even more precise assays such as multiplex RT-qPCR or droplet digital PCR (ddRT-PCR) [2,4,5,6,7]. Such protocols are required for novel disease etiology and effective inpatients treatment [1,2,3,4,5,6]. However, as the COVID-19 pandemic has progressed, mitigation efforts require massive testing [2,4,6,8], ideally linked to comprehensive surveillance frameworks to achieve early virus detection at an ambulatory level for an effective transmission rate reduction and quick intervention and treatment. This preventive scope is critical on the underway shutdown–reopening socioeconomic approach forced by massive contagion, changes in virus prevalence, and new SARS-CoV-2 strains [9]. Success on COVID-19 mitigation under the uncertain ‘new normality’ must rely on surveillance approaches complementing clinical needs and medical developments by providing comprehensive population risk assessments [10,11]. Thus, we pursue this research to develop a safe, low-cost, in-house protocol for SARS-CoV-2 RT-qPCR-based detection using mouthwash–saliva self-collected specimens to achieve clinical and epidemiological purposes under a real-time web environment for effective ambulatory population surveillance.

## 2. Materials and Methods

### 2.1. Ambulatory Patients

This study totalized 115 samples, including 70 mouthwash–saliva (MWS) self-collected specimens and 45 nasopharyngeal swabs (NPS). The experiment was conducted on ambulatory volunteers granting written and signed informed consent, for both, sampling and App baseline data provided, agreeing to be used for research purposes. Anonymity and privacy were assured to participants by handling only ID data and the use of dedicated servers restricted to this research. A privacy notice was also provided through the App. For 3- and 5-year-old children, both parents provided written consent only for sampling. In addition, the Research Technical Committee of the Postgraduate College approved the study. Protocol development and optimization were achieved through three successive cohorts from March to June 2020 during the exponential phase of the COVID-19 epidemic in Mexico. Cohorts included 25 asymptomatic, 2 symptomatic, and 43 suspected cases upon epidemiological criteria. Specific symptoms and volunteers per cohort are included in Table 1. The suspected cases attending a dedicated COVID-19 screening clinic were selected and approved by health authorities of CDMX Cuauhtemoc District (JSC 15) for simultaneous NPS and MWS sampling. The NPS specimens, collected on cohorts 2 and 3, were submitted to a blind national reference laboratory to perform the official test accepted by the World Health Organization (WHO) [1,12].

### 2.2. Self-Collected Specimen and SARS-CoV-2 Biological Inactivation

The self-collected specimen consisted of MWS vigorously stimulated with 1 mL of sterile distilled water for 40 s. Individuals were asked to avoid food eating and/or tooth brushing at least 1 h before sampling, keep the mouth closed during the MWS procedure to avoid aerosols [13,14,15], and stick the ID label and close the lid after collecting the total mouth fluids in a 100 mL sterile leak-proof, screw-cap plastic bottle containing 2 mL of lab-prepared viral Hanks’s transport medium [16]. Afterward, bottles were incubated in a hybridization oven, disabling air-circulation (Thermo Hybaid HS9360, Champaign, IL, USA), at 60 °C for 60 min for SARS-CoV-2 inactivation and then aliquoted into 2 mL tubes and storage at 4 °C/≤24 h [17,18]. Cohorts 1–3 specimens were collected at the Postgraduate College and Secretary of Health-CDMX Clinic 6, respectively. Except for Clinic 6 sampling, which required special protection equipment because of the NPS specimen collection [19,20], protective gear included a KN95 mask, face shield, apron, and nitrile gloves. The protocol was developed in a laboratory bio-safety level II at Postgraduate College, adapting general safety guidelines and recommendations [2,19,20]. Supplementary Materials S1 provides experimental settings to optimize the self-collecting procedure and the real-time RT-PCR in-house protocol [2,3,4,21], including primers/probes and gBlocks targeting the virus N-gene region and the human RPP30-gene.

### 2.3. Web and Mobile Technology for Real-Time Surveillance

A surveillance web platform system, built with HTML, Bootstrap, and PHP programming language combined with JavaScript libraries, linked to a mobile phone Java-android application (App) was developed for individual real-time data collection, diagnostic reporting, health and disease monitoring as a critical prevention proof-of-concept. The aim was to integrate clinical and epidemiological data to support automatic algorithms decision-making at the community level to break SARS-CoV-2 transmission chains by screening individual risk within a target population and testing if required (Supplementary Materials S2). The App was used for an initial survey, through assisted or self-assessment, and further daily self-monitoring. To remove bias, survey responses were App programmed as multiple choices. Additionally, clinical variables were harmonized with official guidelines on the diagnosis of COVID-19. Assisted surveys were conducted by members of the research team. An App help menu was also included for that purpose. On first use, the App automatically generates an ID login and queries optional georeference, personal data such as phone number, age, gender, COVID-19 related symptoms, and as well as health, social, family, and occupational risk factors totaling 93 variables as a core baseline database. The baseline data per participant was collected only one time requiring 20–30 min. Daily self-monitoring requested only 14 variables performed in 3–4 min. The ID was also used to label specimens. Individual data was real-time submitted to the web platform. By logging, limited ID data was available at the laboratory web interphase to restrict information on eventual usage scenarios by accredited private or official laboratories. Diagnostic comprehensive data such as finalization date, positive/negative result, protocol, Ct-value, viral copy number, including retesting if needed, were registered on such interphase. In total, six additional variables were added to the core baseline. After compliance on diagnostic criteria, a ‘*positive*’ or ‘*negative*’ automated text message web–phone service (SMS) was submitted to the suspected individual, which in turn is entitled to resend her/his status, and the App downloading link to the family–social–coworkers subcluster at risk for ready digital assessment. An SMS alert was also sent to a physician and health officials accredited to review digitally the data set profile for specific health risk considerations upon positive results and daily clinical self-monitoring. Supplementary Materials S2 provides the general description and pseudocode of nine algorithms developed for surveillance.

### 2.4. Validation of SARS-CoV-2 Detection Protocol and Web–Mobile Application Platform

The SARS-CoV-2 detection protocol and the web–App surveillance system were simultaneously developed, validated, and improved through three independent ambulatory cohorts. A clinical control, along with gBlock_SARS-CoV-2 N_ (Supplementary Materials S1), was included after a positive case was found and validated with the official test. Agreement between binary data (1, positive vs. 0, negative), obtained from MWS and NPS RT-qPCR diagnostic results, was evaluated using 45 paired specimens of cohorts 2 and 3 with analysis of variance (ANOVA), correlation analysis, and an agreement test. Cohort 1 had only MWS specimens used for the in-house protocol development, thus it was excluded from analyses. ANOVA was performed with the Procedure for Generalized Linear Mixed Models (GLIMMIX) and Fisher’s test (*p* < 0.05). Correlation analysis was undertaken using Spearman’s ρ of CORR procedure. The agreement test was assessed with Cohen’s Kappa coefficient (*K*) of FREQ procedure; *K*-values between −1 and 1 were grouped as ≤0 no agreement, 0.01–0.20 none to slight, 0.21–0.40 fair, 0.41–0.60 moderate, 0.61–0.80 substantial, and 0.81–1.00 almost perfect agreement [22]. In addition, an agreement index (AI) [AI = ((MWS true positive + MWS true negative)/(total NPS test − total NPS inconclusive test)) × 100] was created. The SAS software version 9.4 was used for statistical analyses. Validation of the surveillance system assessed the effectiveness of laboratory diagnostic database integration with core baseline survey and real-time communication upon risk algorithms (Supplementary Materials S2). 

## 3. Results

### 3.1. Self-Collected Specimens in Ambulatory Cohorts 

**Figure 1:**
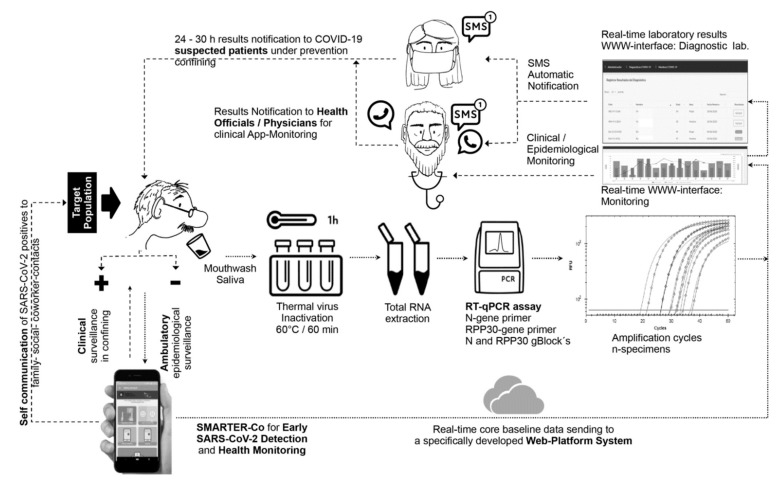
Self-collected mouthwash–saliva (MWS) and RT-qPCR-based protocol for SARS-CoV-2 detection incorporated to a surveillance web–mobile application platform system to integrate real-time epidemiological and clinical core baseline database with laboratory results for decision making on testing, confining, and self-report to family–social–coworker contacts in ambulatory clusters. Dotted lines and small arrows represent the flow process at the first stage, starting from a survey assisted- or self-assessment survey using an android App, followed by MWS collecting specimens if prompted by a risk algorithm. Dashed lines and big arrows represent the flow at the second stage, from real-time communication of testing results to epidemiological and clinical monitoring.

In contrast to 45 NPS sampling, self-collected MWS was successfully done without pain, sneezing, or coughing in 70 individuals distributed in three wide age-range ambulatory cohorts, including one symptomatic 81-year-old male and 3- and 5-year-old asymptomatic children (Table 1, Figure 1). Slight discomfort was observed in a few cases, with throat pain requiring 2–3 pauses to complete the rinse period. Because of that, a 40 s MWS was selected. A safe 2 m distance from collecting individuals limited potential inoculum exposure. MWS critical factors were the transport media volume and mouthwash duration time due to direct effect on the virus load and limiting the virus collection and stimulus of salivary glands (Supplementary Materials S1). An optimal MWS sample, based on yielded RNA, had viscosity, turbidity, density and provided at least 1 mL of specimen. Proper explanation assisted with a photography poster was required to achieve an optima self-collected specimen. Specific sampling procedures tested are included in Supplementary Materials S1.

### 3.2. Validation of SARS-CoV-2 MWS Base Detection Protocol

**Figure 2:**
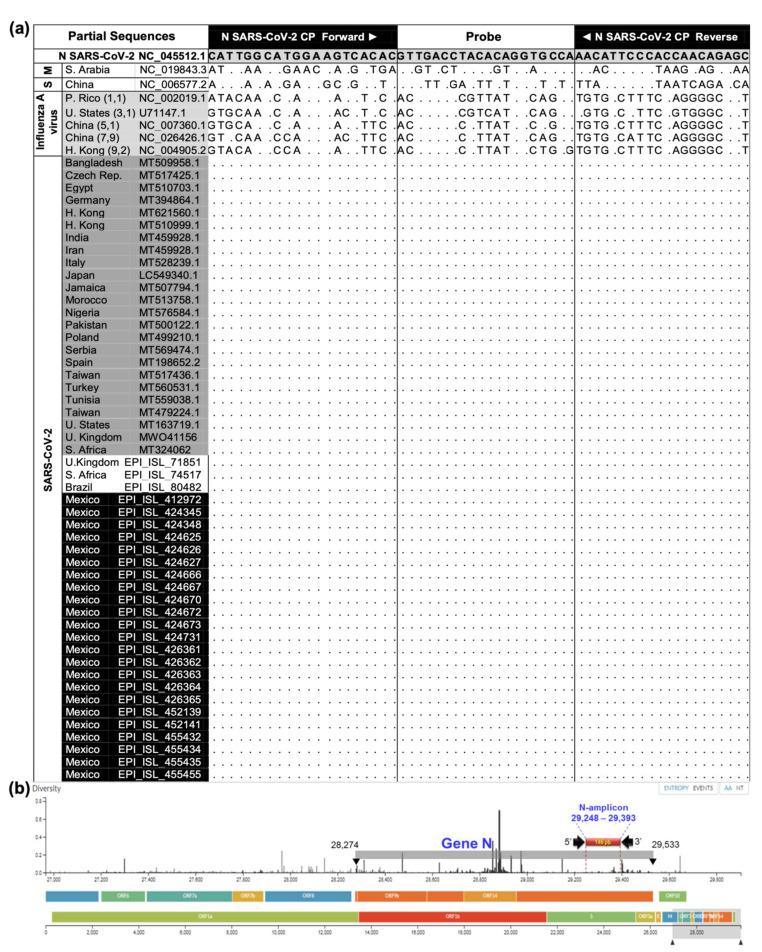
N-gene partial sequence analyses and SARS-CoV-2 genome map. (**a**) CP primer set and probe oligonucleotides generated based on first SARS-CoV-2 sequence (NC_045512.1), and alignments with SARS-CoV-2 (209), 2003 SARS-CoV (S) (1), MERS-CoV (M) (1), and Influenza A virus (5) from NCBI and GISAID. Selected sequences per country represented perfect matches (dots) including strains found in the UK (Alpha), South Africa, and Brazil. (**b**) SARS-CoV-2 full genome, target N-gene location, and binding region selected for the SARS-CoV-2 CP primer set and amplicon size generated. The primer set sequences were located at a high conserved genome. Source: adapted from Nextstrain (https://nextstrain.org/ncov/global?dmax=2020-04-08, accessed on 28 January 2021).

Self-collected MWS specimens of 70 ambulatory individuals and 45 NPS specimens were successfully used to validate the RT-qPCR specificity–sensitivity–efficiency (Table 1; Supplementary Materials S1), and web–App platform applicability (Figure 1). The MWS detection protocol provided similar results to the NPS official test, paired performed in 45 ambulatory individuals of cohorts 2 and 3 (Table 1). The agreement index (AI) was 90% and 95% and Spearman’s correlation of ρ = 0.76 (*p* < 0.0001) and ρ = 0.84 (*p* < 0.0001), respectively. Kappa’s test indicates substantial agreement for cohort 2 (*K* = 0.77, 95% CI 0.46–1.0) and almost perfect for cohort 3 (*K* = 0.83, 95% CI 0.51–1.0). Even though the optimized MWS protocol was improved at cohort 3 by 5% AI units (k = 6 units) by increasing cDNA from 2.5 to 5 μL at the reaction (Supplementary Materials S1), the GLIMMIX showed no statistical difference between MWS and NPS in cohorts 2 (*p* = 1.0) and 3 (*p* = 0.68). Using our ddRT-PCR backup confirmation test (Supplementary Materials S1), two positive (JGS-9600, GMG-5416) and one negative (JDD-4458) RT-qPCR results were discrepant with the official test, confirming a higher sensitivity in our protocol. Ideal RNA concentration (105.5, 52.4, 110.1 ng/μL) and purity (2.2, 2, 2.2 nm) eliminate the sample effect on the detection discrepancy (Table 1). The optimal RNA extraction, with the hot acid phenol method, was used for the three cohorts’ specimens (Supplementary Materials S1; Table 1 S1). Sensitivity was important to avoid false-negative results. These results also confirmed primers’ specificity and stability (Figure 2a,b). Supplementary Materials S1 provides the blasting process performed with 23 Mexican SARS-CoV-2 sequences available during the research development, and 183 selected from 23 countries with high COVID-19 epidemic intensity, including those with the Alpha variant outbreak. Figure 2 shows evidence of specificity and stability on selected sequences.

**Figure 3:**
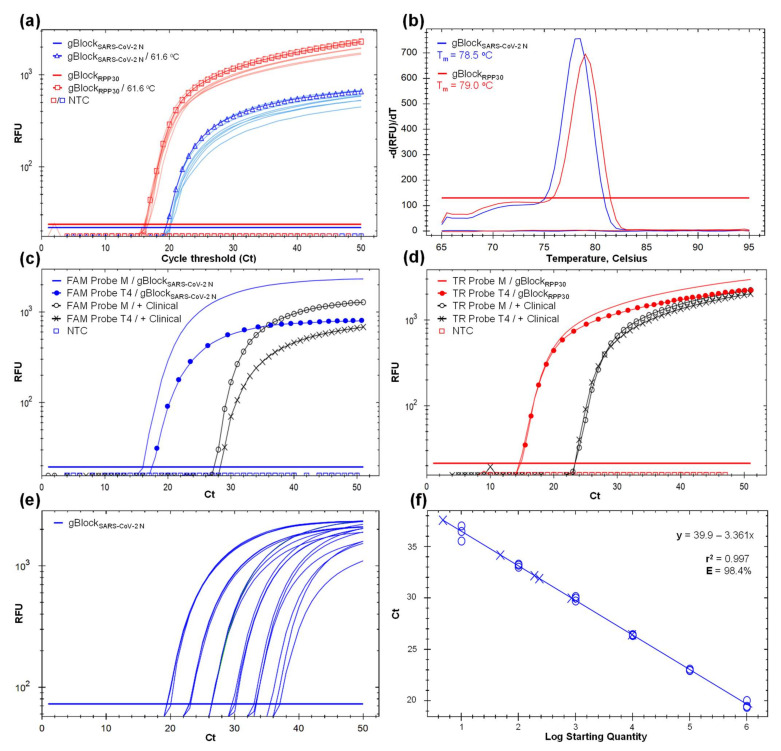
Development and optimization of an in-house SARS-CoV-2 detection protocol based on RT-qPCR and mouthwash–saliva (MWS) self-collected specimen using SARS-CoV-2 CP and RPP30 CP primer sets to target virus N-gene (blue line) and the human endogenous RPP30-gene (red line), respectively. Not target control (NTC) below the threshold line. (**a**) Gradient RT-qPCR amplification curves using the gBlock_SARS-CoV-2 N_ and gBlock_RPP30_ as synthetic template controls and increasing the annealing temperature (T_a_) from 55 to 64 °C. Curves with marks represent the selected T_a_ at 61.6 °C. The *Y*-axis represents the relative fluorescent unit (RFU) variation against the PCR cycle number. (**b**) Melting curve of gBlock_SARS-CoV-2 N_ and gBlock_RPP30_ expressing a single melting point at 78.5 (blue line) and 79 °C (red line), respectively, indicating target specificity. The *Y*-axis indicates the negative first derivative of RFU generated by the reporter during PCR amplification. (**c**) Amplification curves of gBlock_SARS-CoV-2 N_ and positive clinical control (AMB-3661) as templates using a FAM probe synthesized by Macrogen (M) and T4 Oligo (T4). Earlier amplification and optimal fluorescent signal were obtained with M´s oligonucleotides. (**d**) Amplification curves of gBlock_RPP30_ and positive clinical control (AMB-3661) as templates using a Texas Red (TR) probe synthesized by M and T4. Analogous amplification curves and RFU indicates similar probe performance. (**e**) Standard curves with six 10-fold dilution series from 1.016 × 10^6^ to 1.016 × 10^1^, with three replicates per concentration, based on gBlock_SARS-CoV-2 N_ as a template. (**f**) Fitted linear regression of dilution series (r^2^ = 0.99) and amplification efficiency (E = 98.4%).

**Figure 4:**
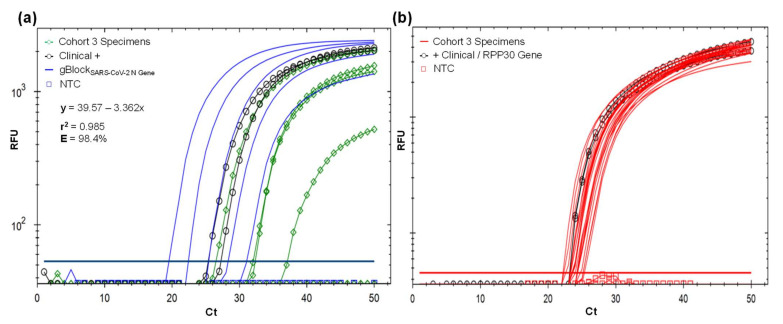
(**a**) Validation of the SARS-CoV-2 detection protocol using SARS-CoV-2 CP primer set and Macrogen FAM probe with 22 MWS self-collecting specimens provided by volunteers of cohort 3. Complete validation results are in Table 1. Four specimens were amplified in the range of 26.9–37.6 Ct, two positive clinical control CDL-3815 and AMB-3661 with 25.9 and 27.63 Ct, and all five 10-fold dilution factor for gBlock_SARS-CoV-2 N_, with 19.6–31.1 Ct, used for absolute quantification shown in the linear regression. Eighteen specimens and NTC did not amplify. (**b**) Confirmation of optimal sample RNA based on the amplification of human internal control RPP30-gene in all 22 MWS self-collecting specimens of cohort 3 volunteers using RPP30 CP primer set and Macrogen Texas Red probe. RPP30-gene region was also amplified in clinical controls CDL-3815 and AMB-3661.

At optimal RT-qPCR protocol setup (Figure 3; Supplementary Materials S1), applied to cohort 3 (Table 1, Figure 4), four individual specimens were positive. Three symptomatic (15%) and one asymptomatic (0.5%), were amplified in the range of 26.9 Ct (5750 viral copy number (VCN)/reaction) to 37.6 Ct (3.6 VCN). Two ambulatory symptomatic positive controls (CDL-3815 and AMB-3661) amplified at 25.9 and 27.63 Ct and the gBlock_SARS-CoV-2 N_ in the range of 19–36.7 Ct associated to five 10-fold dilution factor, included to verify quantification. Based on these results, our RT-qPCR protocol has a positive detection threshold of 37.6 Ct. Eighteen specimens of asymptomatic individuals and the negative control did not amplify (Figure 4a). All 22 specimens and internal control gBlock_RPP30_ amplified with RPP30 CP primer with 22.4 and 25.5 Ct, confirming that RNA was collected and extracted for effective virus detection if present (Figure 4b). This also avoids false-negative outcomes due to a lack of template.

**Figure 5:**
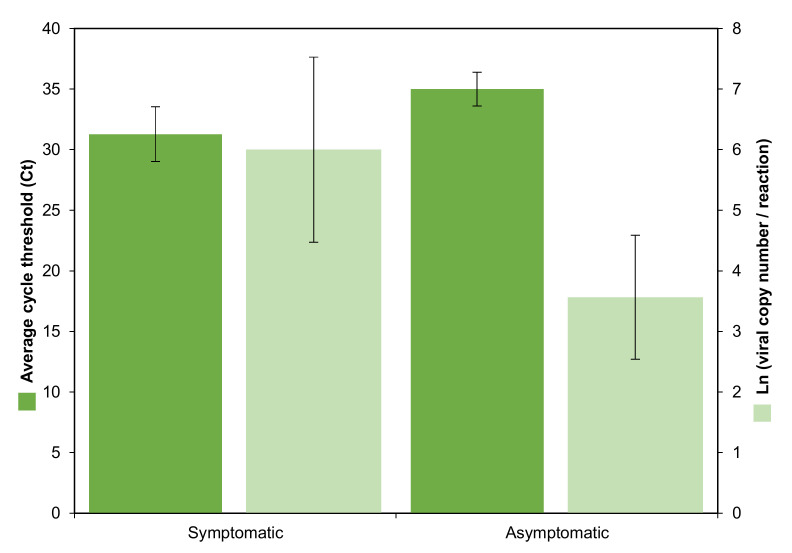
SARS-CoV-2 positive results found in 70 ambulatory volunteers distributed in three studied cohorts. Average relative and absolute quantification (±S.E.M., standard error of the mean) upon cycle threshold (Ct) and viral copy logarithm number per reaction of asymptomatic (5) and COVID-19 symptomatic volunteers (7), were marginally different (normalized *t*-test, *p* = 0.057) and different (*p* = 0.048), respectively.

A probe selection, instead of SYBR Green, and gBlok_SARS-CoV-2 N_, were meant to enhance specific virus load quantification for epidemiological and clinical purposes (Table 1; Figure 5). Including all cohorts, 12/70 (17.1%) were positive in the range of 720–3.08 × 10^6^ VCN/mL (25.8 to 37.6 Ct). The average viral load and cycle threshold were higher, but marginally different upon a normalized t-test (*p* = 0.057), in symptomatic volunteers (7/70, 10%) with 747,294.3 VCN/mL (range 1433.6–3.08 × 10^6^; 31.3 Ct ± S.E.M. 4.5) with respect to asymptomatic cases (5/70, 7%) with 21,767.7 VCN/mL (range 720–77,278; 35 Ct ± S.E.M. 2.8), even with the small positive sample size (Figure 5).

### 3.3. Web and Mobile Technology for Real-Time Surveillance 

**Figure 6:**
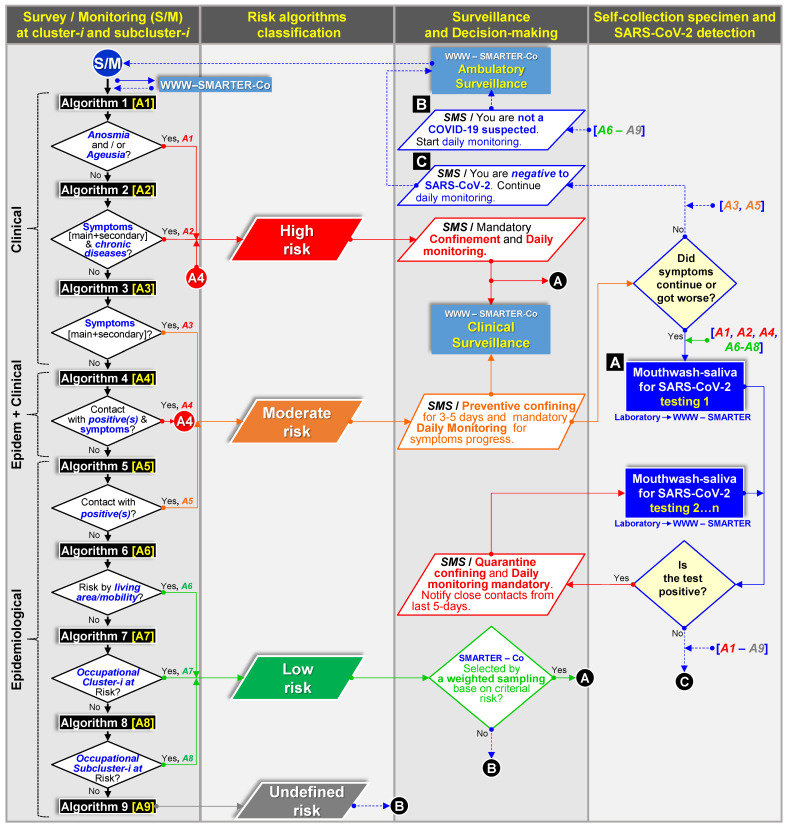
Flowchart of nine automatic algorithms based on clinic [***A*1–*A*3**], clinic-epidemiological [***A*4**], and epidemiological [***A*6–*A*9**] criteria build-in on a web platform–mobile technology system (**WWW–SMARTER–Co**) used for decision-making communication (**SMS, SMARTER-Co**) on testing (**A**), confining, and monitoring (**B**,**C**) to support clinical and epidemiological surveillance of ambulatory population clusters for SARS-CoV-2 prevention (Figure 1, Supplementary Materials S2). Color flows represent a specific infection/contagion risk level.

The App was successfully used for self-assessment survey in an educative-occupational location (cohort 1) and assisted assessment at a clinic open environment using the basic protective gear (cohorts 2 and 3). The 93 data entries, surveyed in a 20–30 min frame, contained demographic (24), epidemiological (36), and clinical (33) variables representing the core baseline. Each survey was real-time submitted to the web platform (Figure 1). A total of 37,351 baseline metadata, including 1800 laboratory entries, were integrated into the database. Table 1 included selected core baseline variables along with testing results. Even though all individuals were selected for NPS and/or MWS sampling to achieve this research objective, as ambulatory surveillance proof-of-concept (Figure 1), baseline metadata was used to automatically fit one of nine risk algorithms upon clinical (3), epidemiological (5) and clinical–epidemiological (1) information, which in turn, these comprised three risk categories towards COVID-19: high, moderate, and low (Figure 6; Supplementary Materials S2). The web platform-App synchronization allowed the end-user upon daily monitoring to receive a prompt for SARS-CoV-2 testing, immediate medical care assistance, and preventive confinement while expecting testing results. These categories were also used for conformity assessment with RT-qPCR Ct-value, NPS test result, and to justify a confirmatory ddRT-PCR test (Supplementary Materials S1). 

A core baseline overview showed significant variability as expected in ambulatory populations. The mean age was 37.4 years (range 3–81), with 61% male. Among those suspected cases (*n* = 13/70), the most common symptoms were sore throat and headache, followed by muscle aches and fever. Shortness of breath was not reported. The period with putative symptoms at sampling was five days on average (range 1–10), and the average contact with confirmed COVID-19 was 0.7 (±1.3) (Table 1). The most-reported chronic diseases were obesity (10%) and hypertension (6%). Restringing to positive individuals (17.1%) (Table 1), the overview exhibits that symptomatic cases were 58.3% (41.7% males) ranging from 24 to 81 years presenting mainly headache and fever for 1–3 days, 43% had diabetics or obesity, and 57% had contact with 1–5 positives. In contrast, 41.7% (25% males) were asymptomatic cases, at a similar age range, and smoking was the leading risk factor (20%) (Table 1). Low-risk individuals (54.7%), supported only on the putative COVID-19 symptoms algorithm, failed to predict low positivity outcomes. Thus, risk factors and epidemiological inputs strengthen decision-making algorithms (Figure 6; Supplementary Materials S2). Real-time SMS diagnostic communication was achieved, avoiding reporting delays (Figure 1). Cohort 1 at the labor location allowed effective daily reporting and prompted preventive confining and testing when required (Figure 6). Additionally, daily digital clinical surveillance was accomplished in three weeks through the positive volunteer ACI-3105 until a negative test (Figure 1).

## 4. Discussion

The novel SARS-CoV-2 prompted effective detection protocols readily adapted from previous coronavirus research [1,3,23]. Reliable protocols were provided for clinical purposes [1,2,3,4,5,6,8,15,24]. However, their application on ambulatory population surveillance has rational and operative drawbacks [6,10,11,13,25]. This research is a proof-of-concept of preventive surveillance based on the integration of two major system components (Figure 1): a suitable in-house RT-qPCR detection protocol using low-risk mouthwash–saliva self-collected specimen, compatible with massive ambulatory testing through broad-spectrum molecular laboratories; and a real-time surveillance web–mobile application platform prototype supported by nine automatic decision-making algorithms (Supplementary Materials S2).

The first component, the RT-qPCR detection protocol, was successfully developed and optimized, assuring specificity, sensitivity, and efficiency of the sampling foundation. Extensive evidence shows that saliva is a reliable COVID-19 diagnostic specimen at a similar level to the NPS and OPS swabbing [8,13,14,15,22,26]. However, our MWS procedure is the first that demonstrates oral fluid efficacy for early, low virus titer detection (≥3.6 VCN/reaction, 37.6 Ct). Two reported mouthwash procedures, conducted with suspected [26] or positive cases [27], had contrasting results being effective only in the latter; perhaps due to limited virus concentration in the 10 mL of sterile water or normal saline used for washing. In this work, MWS was applied in heterogeneous ambulatory cohorts, including asymptomatic and mild-symptom suspected COVID-19 cases without preceding testing. This MWS procedure simulates extensive sampling scenarios at the community level, as required for preventive surveillance. The volume of collected fluids, including respiratory tract virus-laden particles, fewer saliva–ribonuclease, and upper respiratory tissues stimulus may be the explanation for our results [15,22]. These tissues are first exposed to airborne SARS-CoV-2 spreading and are perhaps associated with primary infection [15,28]. Effective mouth washing produces a 1–1.5 mL specimen, mostly by triggering salivary glands, avoiding heavy dilution [26]. SARS-CoV-2 can multiply in epithelial cells lining salivary gland ducts [28], allowing hypothetically, to collect only infective virus particles [22]. On the contrary, the NPS sample may include ‘dead’ particles compromising retesting results on inpatient release [25]. With the 2003 SARS-CoV coronavirus, the detection was accomplished with saliva before lung lesions appeared [29], suggesting that saliva can be responsible for asymptomatic infections and effective air transmission [15,22,28]. In fact, high angiotensin-converting enzyme 2 (ACE2) receptor expression, required for virus infection, was found in salivary glands [30]. Despite these pieces of evidence, swabbing procedures are the standard reference for saliva studies mostly conducted with advanced infected hospitalized patients [8,13,14,15,22,31,32,33]. Nevertheless, saliva samples have been PCR positive regardless of negative NPS result, suggesting higher sensitivity [13,14,33,34]. Even so, saliva may exhibit faster virus titer decline at a late stage of infection, explaining differing results where ribonuclease may be involved [22,32,33,35].

MWS self-collected specimens overcome risks associated with invasive methods and may encourage testing in safer environments such as schools or workplaces [8,15,22,34]. Treatment trials and dynamic virus loading studies [22,35,36] can also benefit due to sensitivity and by avoiding the discomfort of serial retesting on the same individuals. To enhance safety further, the specimen was heat-inactivated [18,37]. Although heating has detrimental detection at prolonged high temperatures and low virus titer on swabbing samples [17,24], our results at 60 °C/1 h using Hanks´s medium proved effective in maintaining nucleic acid properties for at least five days [32], perhaps by ribonuclease heat inactivation. Trizol reagent, as an alternative to heating, has been successfully applied in a laboratory-safe low-cost SARS-CoV-2 OPS protocol detection [2].

SARS-CoV-2 CP primer specificity and sensibility were of primary concern in recognizing virus variability [38,39]. Firstly, we excluded the use of primer combinations suggested on earlier protocols because COVID-19 etiological insight was resolved, and clinical diagnosis was improved [1,2,3,4,25,35]. Cost and time were also accounted for [2,38]. Secondly, to prevent false negatives, the relatively conserved N-gene among sarbecoviruses was selected over S, E, or ORF_-n_ as detection targets [1,3,4,27,38,39]. As SARS-CoV-2 global genomic databases increased (Mexican sequences rose from 28 to 720 by February 2021), primer/probe specificity was successfully confirmed three times in this research with 209 sequences, including virus Alpha strain and related respiratory virus. Still, as with other target regions, N-gene variability may compromise primer stability requiring periodical validation (Figure 2b) [38,39]. It is remarkable that N-gene primers have been associated with RT-PCR diagnostics with similar sensitivity (≤10 copies/reaction) or higher than ORF_-n_ genes (1:10, 1:1.6) and E-gene, either using saliva or other invasive procedures [1,3,24,27]. This was attributed to expressed subgenomic mRNA on infected cells resulting in higher N-gene copies [3]. With 3.6 VCN/reaction (37.6 Ct), this research adheres to N-gene sensitivity but is enhanced by sampling. MWS based protocol outperforms 95% NPS detection agreement and other statistic parameters suggesting early low-titer SARS-CoV-2 detection. Asymptomatic infections were detected in the range of 720–77,278.3 VCN/mL, whereas the NPS official test failed to detect below 1440 VCN/mL (Table 1). Yet, other assay differences may contribute to this result. To our knowledge, there are no SARS-CoV-2 viral load insights on asymptomatic ambulatory cases with non-invasive sampling. However, on symptomatic and saliva procedures, a range from 990 to 1 × 10^10^ VCN/mL was reported [14,15,35]. Synthetized gBlock_SARS-CoV-2 N_ was fundamental for virus titer estimation instead of cell-cultured viral RNA or plasmid DNA not feasible in our laboratory [1,3,35]. The SARS-CoV-2 detection threshold, based on this protocol and sensitivity data, was 37.6 Ct.

The second component successfully incorporates COVID-19 diagnosis on the digital platform system. SARS-CoV-2 detection under the surveillance scope is well-differentiated from clinical goals [40,41]. Our rationality was to account for community contagion with early virus detection, confining, and monitoring for effective prevention [11]. Pragmatically and theoretically, this view is fully recognized [40,41,42]. Moreover, first and further pandemic waves exhibited the health system’s limitations to reach the ambulatory population with sound strategies besides vaccination and the hospital environment [43,44]. Despite that, limited efforts have been directed at comprehensive community surveillance systems integrated into the laboratory and health care networks [45,46]. WHO´s sentinel approach and epidemics traceability web systems lack surveillance community strategies [47,48], whereas extensive mobile applications mostly deal with suspected location-tracking and clinical information [49,50,51]. These fragmented solutions and excessive tools confuse the community and discourage policy- and decision-makers [11]. Our digital development effectively integrates clinical and epidemiological core baseline database with diagnostic results targeting specific ambulatory populations. Basic algorithms, such as induction to preventive and quarantine confining and clinical monitoring, were validated in an educative–occupational cluster with cohort 1 volunteers and patient ACI-3105 (Supplementary Materials S2). Registered physicians may provide digital clinical monitoring and health care considering clinical information automatically updated at the App interphase by patients. This configuration was validated along with a physician. Similarly, although only confirmed with our laboratory workflow, *n*-certified centers can be registered for simultaneous testing. Data entry and effective real-time communication were optimized throughout testing of all cohorts.

The flexible architecture system can include *n*-surveillance community clusters (schools, companies, etc.), optimally including health care networks and vaccination programs covering geographical scenarios for integrative regional risk analysis. Additionally, algorithms can be readily adapted to evolving clinic and epidemic scenarios due to new SARS-CoV-2 variants or mitigation effects. We defined an ambulatory population as structured clusters with explicit functional relations instead of constitutive random events [11]. Our validation, although limited, suggests that clustering surveillance could be more effective. It also adheres to clustering infection patterns and surveillance recommendations for COVID-19 [22,40,41,52,53]. This research provides a proof-of-concept for further development and rational frameworks [42,43,44,45,46]. For instance, to improve situational awareness and extensive mitigation responses, the bidirectional hierarchical communication structure of local, regional, and national health public systems still need to be addressed at the architecture system. However, scaling to a labor environment, as an essential socioeconomic cluster, optimizing its organizational structures may prevent contagion and avoid shutdowns. While writing this paper, we started a pilot implementation of the surveillance system engaging voluntarily 627 employees during partial lockdown due to COVID-19. Preliminary results with 644,491 baseline metadata, including 8126 laboratory entries, confirmed cluster and subcluster surveillance applicability, achieving faster decisions on risk communication, sanitation, confining, and clinical monitoring, allowing continuity of essential activities.

## 5. Limitations

Our surveillance approach focuses on ambulatory prevention of SARS-CoV-2 in community clusters. The architecture of the digital platform was conceived with our experience in web-based surveillance systems and meetings with health authorities. However, we lacked extensive data due to limitations in reaching active labor clusters due to COVID-19 lockdown. Although the algorithm flowchart was fully tested with 37,351 data entries, additional risk algorithms may be needed according to the new COVID-19 epidemic developments and treatments. For instance, vaccine type, administration date, and reaction symptoms were programmed for the baseline database for additional risk calculations but were not validated due to delays in the vaccination program, restricted time frame, and funding constraints. In this work, participants in an educational environment were not reluctant to provide sensitive personal and medical data. This may be different in other cluster environments. Our assumption about the values, emotional ties, and co-responsibility within cluster members to adopt self-monitoring, risk communication, and prevention measures were partially validated and still need further study. For SARS-CoV-2 testing, we assumed that a conventional biosafety level II molecular laboratory in a research institution can perform diagnostic procedures. However, for surveillance in general occupational environments, certified laboratories are required for testing. Our web system was programmed to register *n*-laboratories. This function was not validated due to government regulations. Additionally, we were unable to test the digital system structure to handle *n*-cluster big data on a dedicated server. Effective and sustainable prevention requires synchronous surveillance of the *n*-cluster at the local and regional level.

## 6. Conclusions

This research is a proof-of-concept of preventive surveillance based on integrating an RT-qPCR detection protocol using low-risk mouthwash–saliva self-collected specimen, compatible with massive ambulatory testing, with a real-time surveillance web–mobile application platform prototype supported by nine automatic decision-making algorithms. The surveillance system generated 37,351 baseline metadata, including 1800 laboratory entries during the study. The system accounts for community contagion with early virus detection, confining, and monitoring for effective prevention. The platform requires health policies and institutional frameworks for effective implementation like any surveillance system. Additionally, immunological or RT-qPCR detection protocols, suitable for massive testing, can be promptly adapted to this surveillance system. Accordingly, although the App can be found at Play Store^®^ as SMARTER-Co Version 2.0, it was not conceived as a standalone device for independent use. To our knowledge, this is the first surveillance prototype targeting structured community clusters, and clinical and epidemiological settings. The in-house protocol based on easy, safe specimen sampling and handling, costing USD 13.5 without labor, may allow testing of SARS-CoV-2 in any conventional level II bio-safety, broad-spectrum molecular laboratory using 80% of conventional reagents and supplies. This protocol is the first that demonstrates oral fluids efficacy for early, low virus titer detection at an ambulatory environment, including asymptomatic individuals. Successive COVID-19 pandemic waves, regardless of vaccination and all social–economy efforts, clearly suggest the need for new paradigms towards prevention with digital surveillance as the comprehensive system approach.

## Figures and Tables

**Table 1:** Validation of SARS-CoV-2 in-house RT-qPCR detection protocol with three ambulatory cohorts of symptomatic and asymptomatic suspected COVID-19 cases using mouthwash–saliva (MWS), self-collected specimen, compared to nasopharyngeal swabbing (NPS), and a surveillance web–mobile application platform prototype.

Cohort ^1^	Volunteers /Sex	Age (Years)	Clinical Condition	Days with Sym	Contact Num. with Patients	Ct Value	Viral Copy Number (VCN) Per Reaction	Test Result	Official Result	Agreement Tests	Nucleic Acid Conc. (ng/μL)	Purity RNA 260/230 nm
**1**	12/F	21–55	As	-	0–1	NA	-	N	-	-	50–1835.4	1.8–2.4
**(25) ^2^**	13/M	3–59	As	-	0–1	NA	-	N	-	-	30–255.3	1.7–2.5
	2/F	19, 26	As	-	0–3	36.7, 36.6	9.5,10.2	P	P		29.1–66.9	1.7–2.1
	3/F	29–41	As	-	0	NA	-	N	N		28.9–82.2	1.6–2.2
	1/F	33	As	-	0	NA	-	**N**	**P**	AI = 90%	110.1	2.2
	1/F	45	Sy^Ap^	1–3	2	36.4	11.6	P	P	ρ = 0.76	32	2.01
**2**	1/F	47	Sy^St,Tp^	1–3	2	NA	-	N	N	*K* = 0.77	224	2.2
**(23)**	5/M	36–54	As	-	0–1	NA	-	N	N		67.2–154.4	2.0–2.2
	2/M	26, 40	As	-	0	32.8, 31.3	134.5, 386.4	P	P		39.8–301.7	2.0–2.3
	1/M	81	Sy^An, Ag^	1–3	0	25.8	15,407.8	P	P		288	1.9
	1/M	32	Sy^Fe,He,St^	1–3	1	27.8	4709.7	P	P		285	2.3
	1/M	24	Sy^St^	1–3	0	37.1	7.2	**P**	**N**		52.4	2
	5/M	24–47	Sy^St,Tp^	1–10	0–1	NA	-	N	N		86.1–337.2	2.0–2.3
	12/M	22–67	As	-	0	NA	-	N	N		20.7–312.7	1.8–2.3
**3**	6/F	23–55	As	-	0	NA	-	N	N	AI = 95%	16.4–258.2	1.5–2.2
**(22)**	1/F	27	Sy^He^	1–3	5	32.5	130.3	P	P	ρ = 0.84	157.4	2.2
	1/M	55	As	-	0	37.6	3.6	**P**	**N**	*K* = 0.83	105.5	2.2
	2/M	38, 48	Sy^Fe,He,Ap^	1–3	0–1	32.4, 26.9	138.5, 5750	P	P		66.8–340.7	1.8–2.0

**^1^ MWS** self-collecting specimen. Date: cohort 1 = 13/05/20; cohort 2 = 03/06/20; cohort 3 = 17/06/20. **NPS**: cohorts 2 and 3. Total volunteers are in parentheses. **F** = female, **M** = male; **As** = asymptomatic, **Sy** = symptomatic: Anosmia^An^, Ageusia^Ag^, Headache^He^, Sore throat^St^, Articulations pain^Ap^, Fever^Fe^, and Thoracic pain^Tp^; **N/A** = no amplification; **viral copy number** estimated with model y = 39.57 − 3.36x, r^2^ = 0.99; **N** = negative test, **P** = positive test. All specimens were run in duplicate. The internal control human gene (RPP30) was amplified in all 70 specimens analyzed. **Agreement tests**: AI = agreement index; Spearman’s ρ, *p* < 0.0001; Kappa’s *K*, 95% CI 0.46–1.0 in the second cohort, and 95% CI 0.51–1.0 in the third. **^2^** Numbers in parentheses (*n*) indicate ambulatory volunteers. Cohorts 2 and 3 were paired sampled.

## Data Availability

Due to the nature of this research, participants of this study did not agree for their data to be shared publicly, so supporting data is not available.

## Supplementary Materials

The following supporting information can be downloaded at https://www.mdpi.com/article/10.3390/ijerph19031271/s1, Supplementary Materials S1: Total RNA isolation, cDNA, oligonucleotides-gBlocks design, and real-time RT-PCR. Supplementary Materials S2: Automatic decision making algorithms applied to a web-platform surveillance system: An occupational–academic cluster case.
