# Supplementary material for: Surveillance Web System and Mouthwash-Saliva qPCR for Labor Ambulatory SARS-CoV-2 Detection and Prevention
Source: Int J Environ Res Public Health. 2022 Jan 24;19(3):1271. doi: 10.3390/ijerph19031271 (PMC8835463; doi:10.3390/ijerph19031271)
Supplement: Supplementary file 1 [file ijerph-19-01271-s001.zip › SM2 Automatic algorithms applied web-platform 18Jan22final.pdf]

International Journal of *Environmental Research and Public Health*

MDPI

***Supplement S2. Automatic decision-making algorithms applied to a web-platform surveillance system: An occupational-academic cluster case***

***INPUTs { Web-Platform and App-SMARTER-Co }***

// ***Category 1.*** *Initializing survey and monitoring of the population at cluster-$_i$ and time-$_j$ //*

***$P_n$*** ← Main cluster: Ambulatory population (***ap***) at workspace
***$Am_c$*** ← Subcluster-*n* (Subc): ***ap*** with functional relationships (external or internal at workspace)
***$P_{pt}$*** ← Operative team 1: Certified Physician(s)(***$P_{pt\text{-}1}$***) and laboratory technician(***$P_{pt\text{-}2}$***)
***$P_{ht}$*** ← Operative team 2: Health commission
***$M_t$*** ← Operative team 3: Mitigation and sampling crew
***t*** ← Implementation time ($t_0 \ldots t_j$) of the *Surveillance System [Continuous to End period]*
***$t_0$*** ← Start surveillance
***$t_1$*** ← Time at qPCR results
***$t_j$*** ← Daily monitoring ($t_{0+1} \ldots t_j$)
***$S_u$*** ← Workspace-*i* with *n*-spatial units defining a subcluster ***ap***.
***SA*** ← Survey assistance
***DSm***← Daily self-monitoring from time-$_{0+1}$ to time-$_j$
***CE*** ← Clinical and epidemiological data at survey [90 variables] and monitoring [25 variables]

// ***Category 2.*** *Clinical data of individual-i (i = 1…n individuals at **$P_n$** //*

***$M_s$*** ← Main putative COVID-19 symptoms at individual-*i* [*Fever, Dry cough and Headache*]
***$S_s$*** ← Secondary putative COVID-19 symptoms at individual-*i* [*Body Pain, Throat Pain, Rhinorrhea, Conjunctivitis, Chest pain, Diarrhea, Chills, Nausea, Vomiting or Fatigue*]
***Cd*** ← Chronic diseases [*Hypertension, Diabetes, Obesity, COPD, Smoking, Cancer, HIV, Asthma, Cardiovascular Diseases, Renal or Hepatic Insufficiency & Immunosuppressive*]
***Am*** ← Ambulatory individual-*i* with putative COVID-19 symptoms (***$M_s$*** *and/or* ***$S_s$***)
***$P_{os}$*** ← Ambulatory individual-*i* positive to SARS-CoV-2
***AA*** ← Anosmia or/and Ageusia at individual-*i*
***Ag*** ← Age of individual-*i* [3 to *n*-years old]
***Re*** ← RT-PCR testing result of individual-*i* using ambulatory *self-collected mouthwash-saliva procedure [“ready” or “in progress”]* (**Figure 1**).
***qPCR*** ← SARS-CoV-2 in-house detection protocol with RT-qPCR (**Supplementary Material 1**).

// ***Category 3.*** *Clinical-Epidemiological and Epidemiological data of individual-i (i=1…n individuals at **$P_n$** //*

***$C_{pos}$*** ← Individual-*i* in contact with a SARS-CoV-2 positive(s)
***$S_{subc}$***← *n*-Spatial units with ***ap*** at risk [*Building, floor, division, family house, other*]
***Occ*** ← Occupational ***ap*** at risk [*Student, teacher, public relationships, driver, gardener, etc.,* ]
***Cur*** ← Classroom or laboratory activity of individual-*i*?
***Tt*** ← Transportation type risk [Low=private, Moderate=other or High=public]
***$Z_{risk}$*** ← Risk associated to the living area of an individual-*i* [*Low, Moderate or High*]
***$Sc_{risk}$***← Subcluster ***ap*** risk [*Low, Moderate or High*]
***Mit*** ← Mitigation protocol [*yes or no*]
***Ws*** ← Weighted sampling base on risk criteria [*selected or no-selected*]

***OUTPUTs { Algorithms 1-9 }*** (**Figure 4**)

// *Type 1. Alert risk notification to user through:*
*SMARTER-Co → WWW platform → SMARTER-Co* **{$t_0$; *SMARTER-Co* → *$P_n$*}** *//*

Upon ***low***, ***moderate*** *or* ***high Risk***:
***Confinement:*** *“No required but daily monitoring suggested”, “Preventive confining and daily monitoring”, “Mandatory confinement and daily monitoring” or “Quarantine confining and daily monitoring mandatory”*
***Surveillance “****Ambulatory”* or *“Clinical”*
***Testing*** = *“You were selected for self-collecting mouthwash-saliva specimen for SARS-CoV-2 detection. Please after sampling, start your self-monitoring”; or "You are not a COVID-19 suspect. Please star daily self-monitoring for early warning"*

***Notification$_1$* / SMARTER-Co**: Based on risk at survey and daily monitoring.

*// Type 2. Alert communication - WWW-Platform notify laboratory results to Operative team 1 and 2*
WWW-Platform → SMS / Dashboard **{** $\boldsymbol{t_1}$; ***WWW-Platform / SMS*** $\boldsymbol{P_{pt-1}} \rightarrow \boldsymbol{P_{pt}}$ & $\boldsymbol{P_{ht}}$ **}**
*//*

***Notification₂* / SMS, Dashboard**: Based on testing results from $\boldsymbol{P_{pt-1}} \rightarrow \boldsymbol{P_{pt-2}}$ & $\boldsymbol{P_{ht}}$.

*// Type 3. Alert communication - Operative team 2 notify testing results to* $\boldsymbol{P_n}$
WWW-Platform → SMS **{** $\boldsymbol{t_1}$; ***SMS*** / $\boldsymbol{P_{pt}}$ & $\boldsymbol{P_{ht}} \rightarrow \boldsymbol{P_n}$ **}** *//*

***Notification*** $_{3.1}$ ***/ SMS***: Based on testing results from $\boldsymbol{P_{pt}}$ & $\boldsymbol{P_{ht}} \rightarrow \boldsymbol{P_n}$

***qPCR*** *Positive = "You are positive to SARS-CoV-2. Quarantine confining and Daily monitoring mandatory. Notify close contacts from last 5-days" or*
*Negative = "You are negative to SARS-CoV-2. Continue daily monitoring"*

***Notification*** $_{3.2}$ ***/ SMS***: Based on positive results prompt for clinical monitoring from $\boldsymbol{P_{pt}} \rightarrow \boldsymbol{P_{os}}$

*// Type 4. Alert mitigation - Operative team 2 notify actions to Operative team 3*
WWW-Platform → *SMS* **{** $\boldsymbol{t_1}$; ***SMS*** / $\boldsymbol{P_{ht}} \rightarrow \boldsymbol{M_t}$ **}** *//*

***Notification*** $_4$ **/ SMS**: Based on positive individual(s) prompt for mitigation protocol from $\boldsymbol{P_{ht}} \rightarrow \boldsymbol{M_t}$

*// Type 5. Alert communication - Positive individual notify to contacts at subcluster(s)-n* **{** $\boldsymbol{t_1}$; $\boldsymbol{P_{os}} \rightarrow \boldsymbol{Am_c}$**}** *//*
SMS, @mail, WSP, etc. **{** $\boldsymbol{t_1}$; ***social networks (SN)*** / $\boldsymbol{P_{os}} \rightarrow \boldsymbol{Am_c}$**}** *//*

***Notification*** $_5$ ***/*** **SN**: Based on SMS of positivity and resending to ambulatory closed-contacts prompting for immediate SMARTER-Co downloading and surveying, from $\boldsymbol{P_{os}} \rightarrow \boldsymbol{Am_c}$

*// Type 6, 7 and 8. Patient appointment for SARS-CoV-2 re-testing after mandatory quarantine period*
WWW-Platform → *SMS* **{** $\boldsymbol{t_j}$; ***SMS*** / $\boldsymbol{P_{pt}}$ & $\boldsymbol{P_{ht}} \rightarrow \boldsymbol{P_{os}}$ **}** *//*

***Notification*** $_6$ **/ SMS**: Based on clinical monitoring by patient, and surveillance by Operative team 1 prompt for re-testing at specific date and time appointed to laboratory-*i.*
From $\boldsymbol{P_{pt}}$ & $\boldsymbol{P_{ht}} \rightarrow \boldsymbol{P_{os}}$

***Notification*** $_7$ **/ SMS**: *Positive = "You are still positive to SARS-CoV-2. Continue quarantine confining and Daily monitoring mandatory. Wait next appointment"*
From $\boldsymbol{P_{pt}}$ & $\boldsymbol{P_{ht}} \rightarrow \boldsymbol{P_{os}}$

***Notification*** $_8$ **/ SMS**: *Negative = "You are negative to SARS-CoV-2. Stop quarantine confining, continue preventive daily monitoring. Reinsert to cluster-i"*
From $\boldsymbol{P_{pt}}$ & $\boldsymbol{P_{ht}} \rightarrow \boldsymbol{P_{os}}$

***Initialize SA;***

*//* ***Algorithm 1*** *(Clinical) based on Symptoms of High Risk //*

*if* ***AA*** = "yes"; *and* $\boldsymbol{M_s} \geq 0$; *and* $\boldsymbol{S_s} \geq 0$; *and* ***Cd*** ≥ 0; *then;*
*print* ***Risk*** → *"High"*
*print* ***Confinement*** → *"Mandatory confinement and daily monitoring"*
*print* ***Surveillance*** → *"Clinical"*
*print* ***Testing*** → *"yes"*; *else*;
*do* ***Notification*** $_1$ [*SMARTER-Co* → $P_n$]; *end do;*
*end if;*
*else;*

// **Algorithm 2** (Clinical) based on Symptoms and Chronic disease //

*if* $M_s \geq 2$; *and* $S_s \geq 1$; *and* $Cd \geq 1$; *then*;
*print* **Risk** → *"High"*
*print* **Confinement** → *"Mandatory confinement and daily monitoring"*
*print* **Surveillance** → *"Clinical"*
*print* **Testing** → *"yes"*; *else*;
do **Notification₁** [SMARTER-Co → $P_n$]; *end do;*
*end if;*
*else;*

// **Algorithm 3** (Clinical) based on Symptoms //

*if* $M_s \geq 2$; *and* $S_s \geq 1$; *then*;
*print* **Risk** → *"Moderate"*
*print* **Confinement** → *"Preventive confining and daily monitoring"*
*print* **Surveillance** → *"Clinical"*
*print* **Testing** → *"no"*; *else*;
do **Notification₁** [SMARTER-Co → $P_n$]; *end do;*
*end if;*
*else;*

// **Algorithm 4** (Clinical-Epidemiological) based on Positive contact and Symptoms //

*if* $C_{pos}$ = "yes"; *and* $M_s > 0$; *or* $S_s > 0$; *and* $Cd \geq 0$; *then;*
*print* **Risk** → *"High"*
*print* **Confinement** → *"Mandatory confinement and daily monitoring"*
*print* **Surveillance** → *"Clinical"*
*print* **Testing** → *"yes"*; *else*;
do **Notification₁** [SMARTER-Co → $P_n$]; *end do;*
*end if;*
*else;*

// **Algorithm 5** (Epidemiological) based on Positive(s) contact //

*if* $C_{pos}$ = "yes"; *then;*
*print* **Risk** → *"Moderate"*
*print* **Confinement** → *"Preventive confining and daily monitoring"*
*print* **Surveillance** → *"Clinical"*
*print* **Testing** → *"no"*; *else*;
do **Notification₁** [SMARTER-Co → $P_n$]; *end do;*
*end if;*
*else;*

// **Algorithm 6** (Epidemiological) based on living area/mobility risk //

*if* $M_s \geq 0$; *and* $S_s \geq 0$; *and* $Co \geq 0$; *and* **Occ** = *"Student"*; *and* **Tt** = *"High"*; *and* $Z_{risk}$ = *"Moderate"* *or* *"High"*; *then;*
*print* **Risk** → *"Low"*
*print* **Confinement** → *"No required but daily monitoring suggested"*
*print* **Surveillance** → *"Ambulatory"*; *else;*
*if* **Ws** = "yes"; *then;*
*print* **Testing** → *"yes"*; *else*;
do **Notification₁** [SMARTER-Co → $P_n$]; *end do;*
*end if;*
*else;*

```
// Algorithm 7 (Epidemiological) based on Occupational Cluster-i at Risk //

            if Ms ≥ 0; and  Ss  ≥ 0; and  Co ≥ 0; and Occ = "Worker"; and Ag ≤ 37 ; and Scrisk = "Moderate"
            or "High"; and Zrisk = "Moderate" or "High"; then;
            print Risk → "Low"
            print Confinement → "No required but daily monitoring suggested"
            print Surveillance → "Ambulatory"; else;
      if Ws = "yes"; then;
            print Testing → "yes"; else;
                              do Notification1 [SMARTER-Co → Pn]; end do;
      end if;
      else;

// Algorithm 8 (Epidemiological) based on Occupational Subcluster-i at Risk //

      if Occ = "Teacher"; and Sclus = "Yes"; and Cur  = "Yes"; then;
            print Risk → "Low"
            print Confinement → "No required but daily monitoring suggested"
            print Surveillance → "Ambulatory"; else;
      if Ws = "yes"; then;
            print Testing → "yes"; else;
                              do Notification1 [SMARTER-Co → Pn]; end do;
      end if;
      else;

// Algorithm 9 Undefined risk //

      if Ms = 0; and  Ss  = 0; and Co = 0; Cpos = "yes"; and Scrisk = "Low"; and Zrisk= "Low"; and Sclus = "No"; and
        Tt= "Low"; then;
            print Risk → "Undefined"
            print Confinement → "No required but daily monitoring suggested"
            print Surveillance → "Ambulatory"
            print Testing → "no"; else;
                              do Notification1 [SMARTER-Co → Pn];
      end if;
            else;
                  while t = "Continuous";
                        repeat DSm;
                        until t = "End period";
end SA;
            else;

//  Notification of decision-making algorithms  through WWW-Platform //

                  if Re = "ready"; then;
                              do Notification2 [SMS, Dashboard / WWW-Platform → { Ppt & Pht }];
                              end do;
                              do Notification3.1 [SMS / WWW-Platform {Ppt → Pn}]; end do;
                  if qPCR = "Positive"; then;
                  print Risk → "High"
                  print Confinement → "Quarantine confining and daily monitoring mandatory";
                  print Surveillance → "Clinical"; else;
                              do Notification3.2 [SMS / WWW-Platform {Ppt → Pn}]; end do;
                              do Notification4 [SMS / WWW-Platform {Pht → Mt}]; end do;
                              do Notification5 [SMS, @mail, WSP {Pos → AmcK  (SN)}]; end do;
                  end if;
                        else;
                              while qPCR = "Positive";
                                    repeat print Risk → "High"
```

```
                        print Confinement → "Quarantine confining and daily
                              monitoring mandatory"; else;
                     do Notification6 [SMS / WWW-Platform {Ppt & Pht → Pos}];
                     do Notification7 [SMS / WWW-Platform {Ppt & Pht → Pos}];
                       end do;
                until qPCR = "Negative";
            else:
            if qPCR = "Negative"; then;
                     do Notification8 [SMS / WWW-Platform {Ppt & Pht → Pos}];
                     end do;
    else;
        while t = "Continuous";
            repeat DSm;
            until t = "End period";
end;
```
