# Supplementary material for: Surveillance Web System and Mouthwash-Saliva qPCR for Labor Ambulatory SARS-CoV-2 Detection and Prevention
Source: Int J Environ Res Public Health. 2022 Jan 24;19(3):1271. doi: 10.3390/ijerph19031271 (PMC8835463; doi:10.3390/ijerph19031271)
Supplement: Supplementary file 1 [file ijerph-19-01271-s001.zip › Supplement S1 Total RNA isolation cDNA 18Jan22final.pdf]

International Journal of
Environmental Research and Public Health

MDPI

Supplement S1. Total RNA isolation, cDNA, oligonucleotides-gBlocks design and real-time RT-PCR

Objective: To describe the experimental settings to optimize the self-collecting procedure and the real-time RT-PCR in-house protocol, including primers/probe and gBlocks targeting the virus N-gene region and the human RPP30-gene.

The SARS-CoV-2 detection rationality applied to surveillance

The surveillance system to target ambulatory populations integrating a SARS-CoV-2 diagnostic protocol and a web-mobile application platform was developed based on the following rationality grounds: **a**). should be suitable for any bio-safety level II molecular laboratory outside the medical field; **b**). must be an in-house RT-qPCR assay using conventional reagents to lower cost and prevent shortcuts on commercial kits; **c**). has to be a self-collected specimen and laboratory-safe to avoid restrictions on qualified healthcare personnel and infrastructure; **d**). based on our research experience on saliva-glandular virus-load detection on insect vectors [1], we assumed that human mouthwash-saliva (MWS) could be a reliable specimen for detection; **e**). due to failing reports on primer specificity [2,3], oligonucleotides ought to be generated based on a conserved virus genome region; **g**). a gBlock targeting such region must be developed to overcome restrictions on clinical controls; and **h**). a web-mobile application platform prototype will provide support for real-time communication, and clinical and epidemiological monitoring.

Total RNA extraction from MWS specimen

The total amount of RNA per specimen was extracted by using 800 µl of a previously homogenized sample. The sample was centrifuged for 2 m at 13,500 rpm at room temperature. A total of 300 µl of supernatant was discarded and the hot acid phenol protocol was followed [4]. All samples were processed within 24 h after the specimen was collected. To assure that heat virus inactivation did not affect the nucleic acid structure [5,6], RNA concentration and purity were determined with a NanoDrop spectrophotometer 2000 (Thermo Fisher Scientific Inc., Waltham, MA, USA) by calculating the optical density ratio at 260/280 and 260/230 nm wavelengths. Likewise, the nucleic acid integrity was verified by electrophoresis in 1% agarose gel. The effect of Hanks´s transport

medium volume, temperature, and period of storage effect on RNA specimen stability were also evaluated by assessing concentration and quantity after completing specific experimental settings on 24–120 h frame (Table 1 S1).

Total RNA extraction of MWS specimen was accomplished with the hot acid phenol method and CTAB. The former was chosen over CTAB due to faster processing and better nucleic acid integrity (Table 1 S1). A critical factor was the specimen amount used for RNA extraction due to direct effect on the virus load. Even though 1000 μl of specimen yielded higher RNA concentration, 800 μl was selected to fit the volume capacity at the reaction (Table 1 S1; Figure 1a,b S1).

Table 1 S1. Factors, variables and conditions assessed to develop an optimized SARS-CoV-2 detection protocol based on RT-qPCR and mouthwash-saliva (MWS) self-collected specimen.

Factor	Element/ Condition	Volume[1]	Temperature (°C)	Time	Concentration
**Specimen**	Mouthwash	**1ml**	**25**	60 s	-
				**40 s**	
				30 s	
	Collecting time	-	**25**	**9 – 12 a.m.**	-
				15 – 18 p.m.	
	Bottle external disinfestation	**Rinse**	**25**	10>10>10 s	Soap> 2%NaOCl>70% ethanol
				**10 s**	**70% ethanol**
**Hanks´s Transport Medium (TM)**	TM in the collecting bottle	**2 ml**	**4 – 8**	**0.5 – 3 h (cohort 1)**	-
		3 ml			
		5 ml		**3.0 – 7 h (cohort 1,3)**	
	TM + specimen at cool storage	**2 ml**<br>3 ml<br>5 ml	**4**<br>*−20*	**24 h**	
				48 h	
				120 h	
**RNA extraction**	CTAB 2%	800 μl	−20	-	Isopropanol + Ammonium acetate 7.5 M
	Hot acid phenol	**800 μl**	**−20**<br>*−80*		
		1000 μl	−20		**Ethanol 96% + A. sodium 3M (2.5: 0.10 / v:v)**
**Reverse Transcription**	Total RNA	**2.5 μl**	-	-	-
		5.0 μl	-	-	-
**qPCR**	Primers<br>Probes	-	55 - 64 (**61.6**)	-	300 - 900 nM (**900**)<br>200 - 900 nM (**500**)
	cDNA	2 – 5 μl<br>(**5.0 μl, N**)<br>(**2.4 μl, RPP30**)	-	-	-
	$gBlock_{SARS\text{-}CoV2\ N}$<br>$gBlock_{RPP30}$	**5.0 μl**<br>**2.4 μl**	-<br>-	-<br>-	10 concentrations at 1:10 dilution factor from 1.016×10^0 –1.016×10^9 (**1.016×10^1 – 1.016×10^6**)

[1]Specifications in bold were optimal for each factor/variable/condition. Specifications in italic were also effective and optional. Volume at specimen factor refers to distillated sterilized water. **TM** was prepared in laboratory; time refers to period from collecting to thermal virus deactivation. Primers and probes as Table 2 S1. gBlocks: **N**= nucleocapsid protein gene; **RPP30**= human internal positive control. Gradient qPCR was performed at the indicated temperature to estimate the annealing temperature.

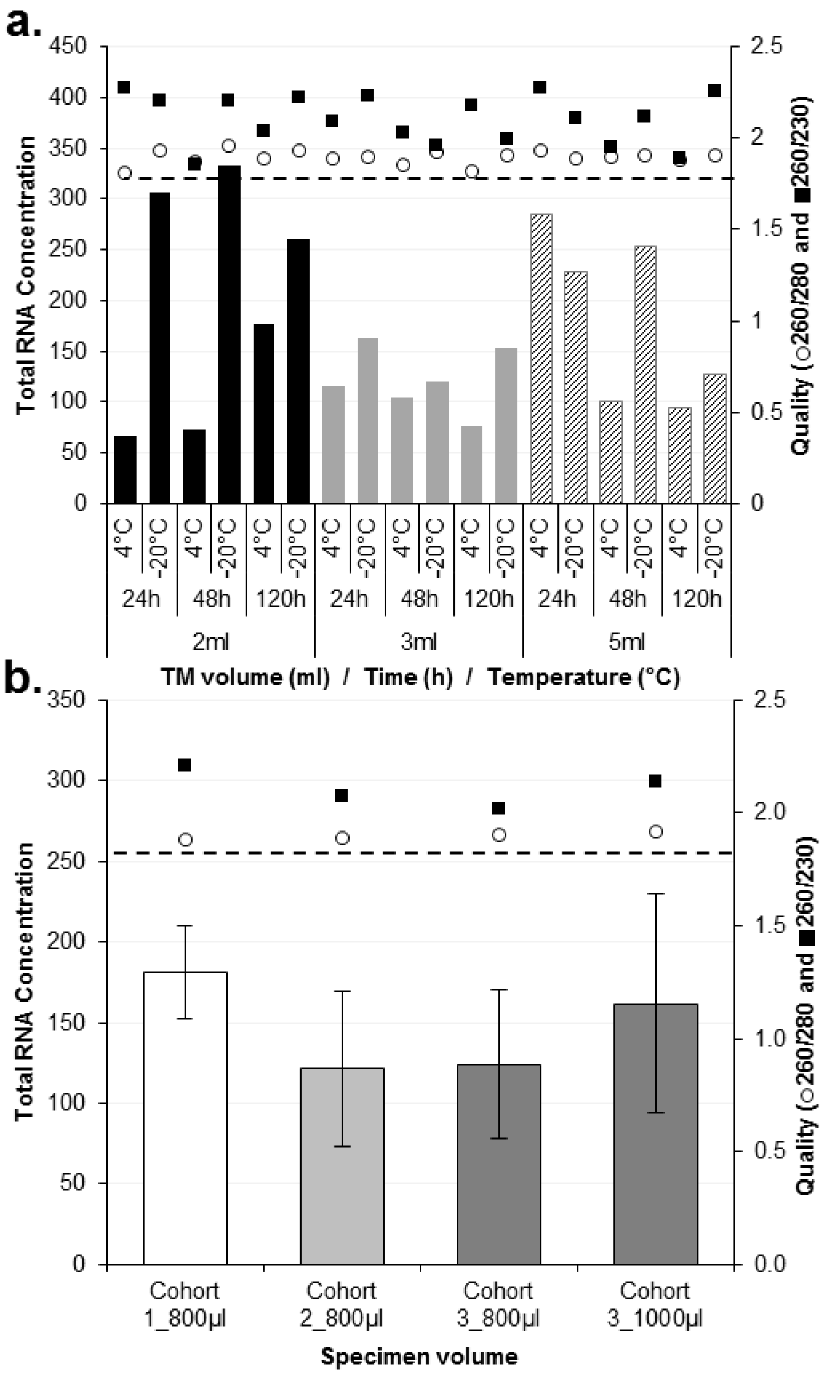

Figure 1 S1. Concentrations (ng / µl) and optical densities ratio at 260/280 and 230/260 of total RNA extracted from a MWS specimens. **a** Effect of TM volume (ml), storage period (h) and temperature condition (°C) on quantity and purity of total RNA extracted with the hot acid phenol method using 800 µl specimen of a confirmed positive volunteer (AMB-3661). **b** Average concentration and optical densities ratio of total RNA extracted with the hot acid phenol method using 800 and/or 1000 µl specimen from all volunteers of cohorts 1 - 3. Dashed line presents the minimum purity threshold at 1.8 optical ratio. Based on error bars there are not statistical differences. Actual values are disaggregated in Table 1 at the main paper.

During the MWS procedure, solid debris such as epithelial tissue and blood in the sample, the time of sample was collected either in the afternoon (cohort 1) or early morning (cohorts 2 and 3), did not affect the quantity and quality of RNA (Main paper, Table 1). This favors the development and optimization of a diagnostic protocol without operational restrictions that hinder its implementation during sample collection and laboratory processing. Additionally, immediate nucleic acid extraction, or after specimen storage, was not significant in RNA isolation. Storage temperatures combined with TM volumes were assessed with a positive case (AMB-3661) showing that 2 ml, plus the collecting specimen, was able to keep RNA stable for up to 120 h at 4 °C or −20 °C. Yet, with a threshold above 1.8 optical density ratio, RNA quality was less affected than concentration (Fig. 1a). Although −20 ˚C storage allowed extractions with higher RNA concentration, 4 ºC was selected to fit common equipment. Total RNA integrity was confirmed by visualization of two bands per sample corresponding to the ribosomal subunits (rRNA). However, due to RNA concentration heterogeneity among samples, difficulties to observe the double bands, and being time consuming, the optimum protocol included the $gBLOCK_{RPP30}$ and RPP30 CP primer as a primary option to confirm effective extraction procedure and RNA stability provided the sample amplification (Table 2 S1). This option may rule out a false negative due to virus template absence in the sample. For validation purposes, samples with low quality and quantity of total RNA extracted were reprocessed. In all cases, the control endogenous RPP30 was amplified between 22.4 and 29.1, regardless of the quality and concentration of total RNA which was in the range of 16.5–1835.4 ng / µl (average 201 ng / µl) (Main paper, Table 1; Figure 1b S1).

cDNA synthesis

cDNA two steps synthesis was run with 2.5 µl and 5 µl of total RNA for optimization purposes. At the first step, a reaction mix of 10.5 µl of nuclease-free water, 6.75 ng of Oligo (dT) 15 Primer (Promega) and 2.5 µl (or 5.0 µl) of total RNA was incubated at 85 °C for 3 min in a Bio-Rad T100 thermocycler. At the second step, 0.5 mM dNTP Mix, 1X buffer-RT, 5.4 U M-MLV Reverse Transcriptase and 0.5 U RNAsin, all from Promega Corp. Madison, WI USA, were added to the reaction. The final 19 µl cDNA synthesis reaction mixture was incubated at 44 °C for 60 min, followed by enzyme inactivation at 92 °C for 10 min. The Oligo (dT) 15

Primer was selected to cover the whole RNA genome allowing possible use of cDNA with additional target virus genes if the protocol update was required. For the cDNA synthesis procedure, 2.5 µl of total RNA was preferred over 5.0 µl because it optimized the M-MLV Reverse Transcriptase reaction.

Primers design for SARS-CoV-2 and human endogenous gene, probes and gBlocks

Primer design and optimization in general adhere to guidelines previously reported for PCR-base SARS-CoV-2 detection [2]. The first step was to select the conserved region encoding the nucleocapsid structural protein (N-gene) as the target gene in the SARS-CoV-2 genome for specific virus detection. This region has relatively low amino- and nucleic-acids mutation frequency in comparison with ORF_{-n} (https://bigd.big.ac.cn/ncov/variation/annotation, accessed on 26 February 2020), a region commonly used in SARS-CoV-2 detection protocols [2,7,8].

As a second step, the first publicly available full SARS-CoV-2 sequence related to a patient from the epidemic outbreak in China [7,8], and the associated N-gene in the coding sequence CDS 28274–29533, were retrieved from the NCBI Reference Sequence Database (https://www.ncbi.nlm.nih.gov/nuccore/ NC_045512.1, accessed on 15 March 2020). At the initial stage of this research, no Mexican sequence was available to be considered. The N-gene was used as a template to design the primer set and probe. Similarly, the full RPP30 human gene sequence was retrieved from NCBI (https://www.ncbi.nlm.nih.gov/nuccore/NC_000010.11, accessed on 15 March 2020) to design a primer sets and probe to target this gene in the human-RNA specimen as an internal positive control for nucleic acid quality [3]. Primers and probes were designed with the Primer3 tool (http://bioinfo.ut.ee/primer3-0.4.0/, accessed on 25 March 2020) using recommended selection criteria [2]. The synthesis was delivered by Macrogen Inc. (Seoul, Korea). A second set was obtained from T4 Oligo Co. (Guanajuato, Mexico).

In addition, one common synthetic template control was designed with 299 bp cDNA fragment containing all binding sites of both primers corresponding to the RNA sequence for SARS-CoV-2 N-gene (156 bp) and RPP30-gene (143 bp) named $gBlock_{SARS\text{-}CoV\text{-}2\ N}$ and $gBlock_{RPP30}$, respectively. The gBlock was synthesized by IDT Inc. (Integrated DNA Technologies, Inc. Coralville, IA, USA)

and copy quantification was determined with a real-time PCR calculator (http://scienceprimer.com/copy-number-calculator-for-realtime-pcr).

The third step was to validate specificity of primers, probes and gBlocks sequences in silico with Blast-NCBI program (https://blast.ncbi.nlm.nih.gov/Blast.cgi, accessed on 31 March 2020). Although N-gene is highly conserved in the sarbecovirus sub-genus [8], which includes the 2003 SARS-CoV [3], due to recognized virus mutability (https://www.gisaid.org, accessed on 26 February 2020), SARS-CoV-2 specificity was also tested upon a broader sequences availability, by aligning 91 virus genomes from 23 countries, including the 26 available for Mexico, using the multiple sequence alignment program MAFFT version 7.4 (https://www.ebi.ac.uk/Tools/msa/mafft/, accessed on 10 July 2020), followed by a colored visualization interphase (https://www.megasoftware.net/, accessed on 10 July 2020). Reference sequences of 2003 SARS-CoV (1), MERS-CoV (1) and Influenza A virus (5) were also selected from NCBI. The latter, although not closely related to SARS-CoV-2, was included due to known co-infection. SARS-CoV-2 sequences were selected with epidemiological criteria such as virus outbreak origin, spreading intensity, mortality rate, touristic activity and labor mobility between Mexico and USA. At the time of preparing this manuscript, three apparently highly contagious SARS-CoV-2 strains were detected in UK, South Africa, and Brazil, being official by the WHO on December 14 and 18, 2020 and January 9, 2021, respectively. Thus, 8, 85, and 25 sequences obtained from GISAID, respectively (https://www.gisaid.org, accessed on 28 January 2021), were readily aligned for primer, probe and $gBlock_{SARS\text{-}CoV\text{-}2\ N}$ specificity test. Table 2 S1 includes oligonucleotides designed and Figure 2 (Main paper) shows the N-gene sequence analyses.

Table 2 S1. Primers, probes and gBlocks specifications used to target SARS-CoV-2 N-gene and human RPP30-gene for a SARS-CoV-2 RT-qPCR-based detection protocol.

Primer Name	Target Genome / Gene	Primer 5'- 3' Forward	Primer 5'- 3' Reverse	Probe 5'- 3'	Amplicon /Sequence (bp)
SARS-CoV-2 CP	SARS-CoV-2/ Nucleocapsid protein gene	CATTGG-CATGGAAGTCACAC	GCTCTGTTGGTGG-GAATGTT	FAM-GTTGACCTACA CAGGTGCCA-BHQ1	146
RPP30 CP	*Homo sapiens* / Ribonuclease P protein subunit p30 gene	TTGTTTGTTGGCCCCTC-TAC	CATCAGCACTGGCAAGA-GAA	TR-ACTTAAGCCAGGA CCCCTGT-BHQ2	133
**gBlock 5'- 3'**					
[1]gBlock$_{SARS\text{-}CoV\text{-}2\ N}$	**TCGCG**CATTGGCATGGAAGTCACACCTTCGGGAACGTGGTTGACCTACACAGGTGCCATCAA-ATTGGATGACAAAGATCCAAATTTCAAAGATCAAGTCATTTTGCTGAATAAGCATATTGACGCATACAAAA CATTCCCACCAACAGAGC**CTAAA**				156
gBlock$_{RPP30}$	**TGAGA**CATCAGCACTGGCAAGAGAAATGTCTGTGTTGTAGATGTTTCACTTGGAAGAAATT-GAAGGACCCTGAGCCTTAAAAGTCTGACAAACTTAAGCCAGGACCCCTGTGGGGAAGGTAGAGGGGCC AACAAACAA**GATTG**				143

[1]Subscript name in gBlocks represents the target gene; gBlock sequence had 5 nt at the fragment extremes, indicated in bold, to assure effective restriction enzymes cut; FAM: 6-carboxyfluorescein; BHQ1: Black Hole Quencher 1; BHQ2: Black Hole Quencher 2

Real-time RT-PCR optimization

To optimize the in-house SARS-CoV-2 RT-qPCR-based detection protocol, several key conditions were assessed through a series of experimental settings (Table 1 S1). Annealing temperature, melting curve, and curve shape associated with lower cycle threshold (Ct) were used to select the best assay setup [2-4]. Experimental and validation settings were conducted with duplicate samples and run with nuclease-free water. For testing SARS-CoV-2 CP and RPP30 CP primers efficiency respective to their gBlock template, monoplex gradient RT-qPCR was performed by increasing annealing temperature (T_a) from 55 to 64 ^oC and combining different primers and probes concentrations according to Table 1 S1. Amplification was carried out on CFX96™ Real-Time System (Bio-Rad Laboratories, Hercules CA. USA), using the SsoAdvance™ Universal Probes Supermix (Bio-Rad Lab. Hercules CA. USA). To verify the optimum PCR conditions, two additional gradients RT-qPCR were run with 10 µl of SsoAdvanced Universal SYBR Green Supermix (Bio-Rad), 900 nM per primer and 5 µl or 2.4 µl according to gBlock (Table 1 S1). The optimum RT-qPCR setting has a final 20 µl reaction volume per assay: 10 µl of SsoAdvanced™ supermix for probes (Bio-Rad), 900 nM primer, and 500 nM probe labeled with FAM (N-gene) or Texas red (RPP30-gene) plus 5 µl or 2.4 µl of cDNA-specimen for virus and human target genes, respectively. The gBlock$_{SARS\text{-}CoV\text{-}2\ N}$ (5 µl) and gBlock$_{RPP30}$ (2.4 µl), as positive controls and nuclease-free water, as negative control

were included. The thermocycling program was optimized to a common sequence for both assays at 55 °C for 10 min, 95 °C for 2 min, followed by 50 cycles of 95 °C for 5 s and 61.6 °C for 45s (Main paper, Figure 3a-e). The MWS RT-qPCR protocol was ready adapted to ddRT-PCR (Droplet Digital PCR) using the Bio-Rad supermix kit (Bio-Rad Laboratories, Hercules CA. USA). Due to the sampling processing cost, this protocol was only used for confirmatory purposes on positive specimens with low virus titer detected with RT-qPCR.

Standard curve for real-time RT-PCR efficiency

To quantify virus copies present in the reaction sample and to accurately determine the detection limit of SARS-CoV-2, a gBlock $_{\text{SARS-CoV-2 N}}$ dynamic range was calculated with six 10-fold serial dilutions, each in triplicate, ranging from $1.016x10^6$ to $1.016x10^1$. The log-serial dilutions quantities against Ct values were fitted to a linear regression [8]. Determination coefficient r^2 and amplification efficiency (E% = $[1\text{-}10^{[-1/slope]}]100$) were determined with CFX Manager Software (Bio-Rad). The RNA copy number was calculated from the Ct values using the model generated (Main paper, Figure 3F).

References S1

1. Loeza-Kuk, E.; Ochoa-Martínez, D.L.; Mora-Aguilera, G.; Rivas-Valencia, P.; Gutiérrez-Espinosa, M.A.; Cintra de Jesús, J.; Villegas-Monter, Á.; Arno-Wulff, N.; Perez-Molphe-Balch, E. Detection of citrus sudden death-associated virus and haplotypes of *Citrus tristeza virus* in *Toxoptera citricida* and *Aphis spiraecola* and implication on citrus sudden death in Brazil. *Agrociencia*. **2008**, *42*, 669–678. http://www.scielo.org.mx/scielo.php?pid=S1405-31952008000600007&script=sci_abstract&tlng=en (accessed on 15 March 2020)
2. Park, M.; Won, J.; Choi, B.Y.; Lee, C.J. Optimization of primer sets and detection protocols for SARS-CoV-2 of coronavirus disease 2019 (COVID-19) using PCR and real-time PCR. *Exp. Mol. Med.* **2020**, *52*, 963–977. https://doi.org/10.1038/s12276-020-0452-7.
3. Won, J.; Lee, S.; Park, M.; Kim, T.Y.; Park, M.G.; Choi, B.Y.; Kim, D.; Chang, H.; Kim, V.N.; Lee, C.J. Development of a Laboratory-safe and Low-cost Detection Protocol for SARS-CoV-2 of the Coronavirus Disease 2019 (COVID-19). *Exp. Neurobiol.* **2020**, *29*, 107–119. https://doi.org/10.5607/en20009.
4. Green, M.R.; Sambrook, J. *Molecular Cloning a Laboratory Manual*, 4th ed.; Cold Spring Harbor Laboratory Press: New York, NY, USA, 2012, Volume 1, p. 34.
5. Pastorino, B.; Touret, F.; Gilles, M.; Luciani, L.; de Lamballerie, X.; Charrel, R.N. Evaluation of Chemical Protocols for Inactivating SARS-CoV-2 Infectious Samples. *Viruses* **2020**, *12*, 624. https://doi.org/10.3390/v12060624.
6. Rabenau, H.F.; Cinatl, J.; Morgenstern, B.; Bauer, G.; Preiser, W.; Doerr, H.W. Stability and inactivation of SARS coronavirus. *Med. Microbiol. Immunol.* **2004**, *194*, 1–6. https://doi.org/10.1007/s00430-004-0219-0.
7. Corman, V.M.; Landt, O.; Kaiser, M.; Molenkamp, R.; Meijer, A.; Chu, D.K.W.; Bleicker, T.; Brünink, S.; Schneider, J.; Schmidt, M.L.; et al. Detection of 2019 novel coronavirus (2019-nCoV) by real-time RT-PCR. *Eurosurveillance* **2020**, *25*, 2000045. https://doi.org/10.2807/1560-7917.es.2020.25.3.2000045.
8. Chu, D.K.W.; Pan, Y.; Cheng, S.M.S.; Hui, K.P.Y.; Krishnan, P.; Liu, Y.; Ng, D.Y.M.; Wan, C.K.C.; Yang, P.; Wang, Q.; et al. Molecular Diagnosis of a Novel Coronavirus (2019-nCoV) Causing an Outbreak of Pneumonia. *Clin. Chem.* **2020**, *66*, 549–555. https://doi.org/10.1093/clinchem/hvaa029.
